# Bactericidal, anti-hemolytic, and anticancerous activities of phytofabricated silver nanoparticles of glycine max seeds

**DOI:** 10.3389/fchem.2024.1427797

**Published:** 2024-08-16

**Authors:** K. B. Vijendra Kumar, Kavitha Raj Varadaraju, Prasanna D. Shivaramu, C. M. Hemanth Kumar, H. R. Prakruthi, B. M. Chandra Shekara, Bhargav Shreevatsa, Tanveer A. Wani, K. C. Prakasha, Shiva Prasad Kollur, Chandan Shivamallu

**Affiliations:** ^1^ Department of Chemistry, Bangalore Institute of Technology, Bengaluru, Karnataka, India; ^2^ Bioscience CLIx LLP, Mysuru, Karnataka, India; ^3^ Department of Applied Sciences, Vishveshvaraya Technical University, Chikkaballapura, India; ^4^ Department of Biotechnology and Bioinformatics, JSS Academy of Higher Education and Research, Mysuru, Karnataka, India; ^5^ Department of Pharmaceutical Chemistry, College of Pharmacy, King Saud University, Riyadh, Saudi Arabia; ^6^ Department of Chemistry, KLE Society’s PC Jabin Science College, Huballi, India; ^7^ School of Physical Sciences, Amrita Vishwa Vidyapeetham, Mysuru Campus, Mysuru, Karnataka, India

**Keywords:** soybean silver nanoparticles, MCF-7, anti-breast cancer, antibacterial action, green synthesis

## Abstract

**Introduction:**

Soybean is a rich source of bioactive components with good nutritional support and is easily available. In the treatment of cancer, green synthesis of silver nanoparticles (AgNPs) from plant-based samples has gained attentions due to its potency and feasibility. In the present study, using soybean extracts (GM), silver nanoparticles are synthesized and analyzed for their anticancer potency.

**Methods:**

The synthesized GM-AgNPs were characterized via UV–Vis spectroscopy, Fourier transform-infrared (FT-IR), scanning electron microscopy (SEM), transmission electron microscopy (TEM), and energy-dispersive X-ray (EDX) techniques for further analysis. Antibacterial activity was evaluated using the disc method and anti-hemolysis activity using the *in vitro* method, followed by anticancer property evaluation by cytotoxicity, cell migration, apoptosis, and cell cycle.

**Results and discussion:**

Our results showed that the synthesized GM-AgNPs were spiral-shaped with a size range of 5–50 nm. The antibacterial activity against *Staphylococcus aureus* and *Klebsiella pneumoniae* showed the maximum zone of inhibition at 250 μg/mL in comparison with gentamicin. On exploring the anti-hemolysis efficiency, at 200 μg/mL, GM-AgNPs showed no hemolysis in comparison to the extract which showed 40% hemolysis. On analysis of GM-AgNPs against the breast cancer cell line, the nanoparticles displayed the IC50 value of 74.04 μg/mL. Furthermore, at the IC50 concentration, cancer cell migration was reduced. The mechanism of action of GM-AgNPs confirmed the initiation of apoptosis and cell cycle arrest in the sub-G0/G1 (growth phase) phase by 48.19%. In gene expression and protein expression analyses, Bax and Bcl-2 were altered to those of normal physiology.

## 1 Introduction

In the prevailing situation, the foremost universal problem associated with human health is the spread of infectious diseases from the surrounding environment and the disease triggered within an individual due to changes in their genes. For the former problem, we need to restrict the multiplication of microorganisms like dreadful bacteria, viruses, fungi, protozoa, and algae in various fields, especially in food, therapeutics, bio-medical industries, and health centers (Brito et al., 2022). This concern has become more complex with the emergence of multiple drug resistance (MDR) exhibited by several bacteria. Vulnerability to microbes will lead to conditions like sickness, aches, inflammation, dizziness, throat irritation, and dehydration; therefore, the information pertaining to present microbial implication indicating resistance is mandatory. As per the recent WHO report, there is a great tendency toward antibacterial resistance that we may experience in the post-antibiotic generation, wherein just a slight infection caused by bacteria can prove to be fatal (Banasiuk et al., 2020). Regular chemical antibiotics function through specific pathways, and their frequent use has led to the acquisition of resistance against them. Consequently, we need to look for their replacement which pose minimal side effects and also have the tendency to withstand resistance for a longer duration by various means.

MDR is not just confined to bactericidal activity; however, it is also experienced during cancer because of alterations in drug targets. Despite significant investments to tackle the growing problem of multidrug-resistant (MDR) bacterial strains, it is concerning that the evolution of MDR bacteria is outpacing the development of new antibiotic classes. This urgent issue calls for innovative and less toxic strategies to prevent and combat these resilient bacteria. In modern healthcare, nanomedicine has emerged as a powerful approach to addressing drug-resistant microbes. By leveraging the unique properties of nanoscale materials, nanomedicine enhances drug delivery, targeting, and efficacy, providing a promising solution to the challenges of microbial resistance (Ahmad et al., 2022). Similarly, the concept of MDR extends to cancer treatment, where nanotechnology in drug delivery can significantly improve the effectiveness of cancer therapies (Al Azzam et al., 2024). Cancer is a non-communicable, chronic disease which generally evinces intricacy during the last stage and is the most prominent life-threatening reason for mortality from around the world (Azuaje, 2016; Selim et al., 2020). Cancer is perceived by aberrant benign or malignant tumors which can metastasize to nearby cells. The characteristics of several tumor cells are that they undergo oxidative stress and stimulate cellular redox imbalance, which contributes to oncogenic induction. In addition, the primary phases of mutagenesis, carcinogenesis, and senescence, constituting “oxidative destruction,” are attributable to their enduring alteration of genetic material (Forni et al., 2014). At present, the most prevalent types of cancer due to which we encounter death are breast, lung, prostate, colon, and rectum cancers. Colorectal carcinoma is the most prevalent cancer detected in both genders (Shejawal et al., 2021), whereas breast cancer has a higher prevalence among women. Exposure to asbestos, arsenic, and aflatoxins; tobacco smoking; excessive alcohol consumption; UV and ionizing radiations; infectious microorganisms; *etc*., are the agents which lead to the development of cancer (Whiteman and Wilson, 2016; de Martel et al., 2020). Conventional diagnosis for cancer includes micro-imaging, tissue biopsy, and histopathology; treatments like surgery, radiotherapy, and systemic therapies such as chemotherapy, immunotherapy, and hormonal treatments are accessible, but with some limitations. We require an innovative technology that enables target-specific drug delivery; therefore, imaging process involving nanoparticles is studied considerably as they improve tumor identification in positron emission tomography, magnetic resonance imaging, computed tomography, and photo-acoustic tomography (Jeevanandam et al., 2020).

Nanotechnology is an integrative field of science which has burgeoning interest worldwide, with huge momentum to usher in a nano-revolution (Ghaedi et al., 2016; Halligudra et al., 2022). It contributes to fabrication, processing, and utilization of materials whose size is less than 100 nm. Nanoparticles feature numerous unique applications by virtue of their surface area-to-volume proportion, which lead to their widespread use in sectors concerned with fuel cells, supercritical fluids, renewable energy storage, super capacitors, heavy metal detection, photocatalysis, chemical catalysis, drug delivery, anticancer activity, antimicrobial activity, pharmaceuticals, and food industries (Kim et al., 2001; Pandey et al., 2013; Dharmatti et al., 2014; Kenchappa Somashekharappa and Lokesh, 2021; Shetty et al., 2021). Nanoparticles were employed primarily for preparing various specialized structures like carbon nanotubes (CNTs), epoxy resin-coated CNTs, quantum dots (QDs), polymer-coated Ag, super magnetic iron oxide nanoparticles (SPION), mesoporous silica particles, catalytic metals, metal oxides, carbon QDs, dendrimers, nanofilms, nanofibers, and reinforced nanocomposites (Mousavi et al., 2018). These structured nanoparticles with a homogenous distribution in terms of size, shape, and crystallinity could be achieved using several physical and chemical techniques of preparation, yet their demerits of usage of pernicious chemicals, extensive instruments, tedious and prolonged processes, *etc*., have made biological synthesis a better option, especially for therapeutic applications. For the last 20 years, the top-down methods have been replaced by bottom-up methods for synthesizing metal nanoparticles using numerous plant resources as stabilizing and capping agents on metallic ions, which have resulted in better efficacy (Kiran et al., 2020; Kumar et al., 2022). Moreover, herbal-based drugs have the disadvantage of poor bioavailability, so the use of nanoparticles acts as an optimistic means to prevail in such situations. Although microbial and animal sources are also used for biological synthesis, plant-based products possess additional benefits of easy handling and a long shelf life. Plant extracts are excellent source of enzymes which tend to participate in redox reaction and are responsible for the formation of metal nanoparticles from their bulk counterparts (Bai et al., 2022).

Currently, noble elemental metals like Ag, Au, Pt, and Pd in nanometric measurements are getting appreciable recognition because of their pharmacological applications with full-featured biological properties (Hemlata et al., 2020). From these metals, silver has been used since ancient times for ornamental and several medicinal purposes, such as antibiotics and wound treatment, and now it is also employed in water purifications, bone prosthesis, catheters, cardiac devices, and various other surgical equipment (Franci et al., 2015; Kang et al., 2017). A synergetic outcome of a reaction among free ions of silver converted into nanoparticles and secondary metabolites would generate multi-utilities intended for biomedical applications. AgNPs form the basis for testing tools employed in diagnosing and detecting sensitive biomolecules (Dhand et al., 2016). Additionally, AgNPs, while functioning as antimicrobial entities, have the upper hand over conventional antibiotics by virtue of their diversified action on bacteria, like entering through the cell membrane, perturbing their structural integrity, denaturing enzymes that aids in the transportation of nutrients, and restricting processes of replication, transcription, and translation (Sampath et al., 2022). This active mechanism exhibited by AgNPs is also found to thrive in relation to their anticancer abilities and is supported by several research studies (Sudha et al., 2017; Satpathy et al., 2018). Meanwhile, studies involving cellular behavior during the aforementioned process showed by synthesized nanoparticles are usually less discussed, which we have tried to bring to light in our research.

A highly effective approach involves leveraging natural products (NPs), which are well known for their rich supply of biologically active compounds. The vast chemical diversity found in natural products results in a wide range of biological activities, making them particularly attractive as starting points in drug discovery. With a long history of medicinal use spanning centuries, natural products serve as a valuable reservoir for identifying potential therapeutic lead molecules. This acknowledgment of their diverse utility highlights the significant role that natural products play in the continual advancement of pharmaceutical research and development (Ahamad et al., 2024). Herein, we have accomplished the green synthesis of AgNPs utilizing the extracts of generally used nutraceuticals such as *G. max.* These plant products were chosen based on their availability and known ethnomedicinal properties; in addition, the phytochemicals acquired from them are plentiful resources and serve as potential therapeutic remedies and healers. Soybean (*Glycine max (L.) Merrill*) is a chief oilseed crop of family *Leguminosae*, which is grown for edible seeds and has rich contents of proteins, fibers, phytosterols, and isoflavones. *G. max* is a subtropical plant native to Southeast Asia and has gained fame worldwide as it controls the levels of testosterone, cholesterol, insulin values, weight gain, inflammatory markers, and oxidative stress (Balanescu et al., 2022). The secondary metabolites present in it have confirmed their specific or combined contribution in inhibiting various medical conditions, like cancer, diabetes, inflammation, and heart and blood vessel diseases; in addition, they are regarded as potential antioxidants as they ward off the excessive generation of free radicals in individuals through the process of radical scavenging ability (Choi et al., 2020). Based on the abovementioned information, our investigation was planned to evaluate the role of phytofabricated AgNPs prepared by effortless and reasonable means as antiproliferative agents through apoptosis, and consequently to determine their biocompatible nature.

## 2 Materials and methods

### 2.1 Plant sample collection and extraction

Fresh seeds of G. max were gathered from the botanical garden and preserved at −20°C prior to analysis. Their authenticity was affirmed by plant taxonomist Dr. Ravikumar K of the University of Trans-Disciplinary Health Science and Technology (TDU). The seeds were surface cleansed using distilled water and pat dried using a neat cotton cloth. Then, 40 g of these plant products were blended with 100 mL of double-distilled water, and the filtrate was collected. Then, the filtrate was subjected to water extraction by boiling for a brief period of 20 min to get the seed extract of G. max, and then cooled to room temperature.

### 2.2 Synthesis of silver nanoparticles

For the preparation of green synthesized AgNPs, the optimized concentration ratio of 1:2 with 5 mM of AgNO3 was used. They were kept for continuous stirring at varied temperatures by boiling for 5 h until the confirmation of AgNPs was evidenced by visual changes in color, and it was further validated via UV–Vis analysis. They were cooled to room temperature, and the residue was kept in a muffle furnace at 3,000°C to obtain silver nanoparticles.

### 2.3 Characterization of nanoparticles

One of the most prevalent forms of reflection is its diffuse nature, and so the Rigaku Ultima IV diffractometer with Cu-Kα1 radiation included nickel monochromator at λ = 1.540 Å, a 2° per min scanning speed, and 2θ running between 10° and 70° to interpret the phase (structure) of the crystallite green synthesized nanoparticles. Optical absorbance was determined by employing a PerkinElmer Lambda-750 UV/VIS/NIR Spectrometer with the wavelength ranging from 300 to 800 nm and scanned at 2 nm resolution. The phytochemical groups present on the surface of AgNPs were examined using UTAR Perkin Elmer Spectrum Two model Fourier transform infrared (FTIR) spectroscopy with the wavenumber from 400 to 4,000 cm−1 in the attenuated total reflection (ATR) mode with the diamond crystal and 2.41 reactive index (24 accumulated scans). The structure and surface topology were captured using the Hitachi SU1510 scanning electron microscope (SEM), and mapping of elemental silver existing in the prepared nanoparticles was performed by energy-dispersive X-ray (EDX). Jeol/JEM 2100FC operated with a 300 kV accelerating voltage. LaB6 filaments were used to ascertain the shape and size distribution of GM-AgNPs in transmission electron micrograph (TEM).

### 2.4 Antibacterial activity

The antibacterial activity of green synthesized silver nanoparticles (GM-AgNPs) was assessed using the disc diffusion method for both Gram-negative and Gram-positive organisms, specifically *Staphylococcus aureus* (ATCC 23235) and *Klebsiella pneumoniae* (ATCC 13773). These bacterial strains were chosen based on their regular incidence and pathogenicity. The bacterial cultures were incubated at 37°C ± 0.5°C for approximately 20 h until the cell culture density reached 0.5 McFarland turbidity, at which point they were ready for evaluation (Ben et al., 2022).

For the determination of bactericidal activity, the procedure reported previously was followed with modifications (Mihailović et al., 2019). Warm nutrient agar medium (20 mL) was poured into sterile Petri plates. Once the medium solidified, 100 µL of each bacterial strain was swabbed onto the surface and allowed to stand for 15–20 min. Sterile discs were placed in various concentrations of the GM-AgNPs, ranging from 250 μg/mL to 31.12 μg/mL, for few minutes. As a control, a gentamicin (10 µg) disc procured from HiMedia, India, was placed in the center of each plate.

To facilitate pre-diffusion of the materials loaded in the discs, the plates were placed in an incubator maintained at 37°C ± 0.5°C for 24 h. The presence of clear zones surrounding the disc indicated the antibacterial activity of the prepared nanoparticles. The inhibition zones were quantified using an antibiotic scale, and this assessment was conducted in triplicate to ensure accuracy and reproducibility.

### 2.5 Anti-hemolytic activity

The hemolytic activity (Četojević-Simin et al., 2015) of bare and coated AgNPs was studied in whole blood of two healthy donors. Then, 0.5 mL of each of the three AgNP-tested samples were separately incubated with an equal volume of blood from each of the donors for 24 h in a biological thermostat at 370°C. Blank samples were prepared; distilled water was added as a positive control (PC) and saline as a negative control (NC). After incubation, the samples were centrifuged for 20 min at 2,000 RPM. Hemolytic activity was assessed using the hemolysis coefficient, which was determined spectrophotometrically (Unico 2802, Unico System, United States) based on the optical density of the samples at a wavelength of 415 nm, corresponding to the absorption band of oxyhemoglobin. To measure optical density, 200 μL of the sample was brought up to 6 mL with the saline solution.

### 2.6 Anticancer activity

#### 2.6.1 Cell culture

The cancerous MCF-7 cell line (ATCC HTB-22) was obtained from the American Type Culture Collection (United Sates) and maintained in high glucose Dulbecco’s modified Eagle’s medium (DMEM) in addition to penicillin–streptomycin (100 U/mL), 10% fetal bovine serum (FBS), non-essential amino acids, and Hanks’ salts within a sterile T25 flask. They were allowed to incubate in humidified conditions at 37°C in a 5% CO_2_ atmosphere for 24–48 h until the required number of cells were acquired; otherwise, they could be passaged (Yodkeeree et al., 2010).

#### 2.6.2 MTT assay

The fundamental mechanism under the MTT assay is the conversion of yellow-colored miscible tetrazolium compound of MTT by the reductase enzyme of mitochondria present in viable cells into a purple formazan product, which gets dissolved in dimethyl sulfoxide (DMSO). The detailed procedure as mentioned in the study by Krauze-Baranowska et al. (2014) was followed for the estimation. When the cells attain confluency, they were detached from the surface by trypsinization and centrifuged to count the number of cells. Then, around 3 × 10^4^ cells in 100 µL medium were transferred to each well in a microtiter plate and stowed in incubator with 5% CO_2_ at 37°C for 24 h. The next day, the partial monolayer of cells was formed, and the supernatant was flicked off so as to add 100 µL of varied dilutions of green synthesized AgNPs, after which it was maintained under the same incubation conditions. Furthermore, the upper solution was discarded, and the MTT reagent of 500 µg was pipetted and incubated in dark for 4 h. Later, the product formed was dissolved in DMSO, and subsequently, the absorbance at 590 nm emitted by each sample is recorded using a plate reader (Tecan SPECTRAFluor plus, U.S.). Doxorubicin (100 µM) was taken as standard. Then, the cytotoxicity exhibited by prepared nanoparticles on cancer cells could be determined by their half-maximal inhibitory concentration (IC50) value.

#### 2.6.3 Cell migration assay

Cells (1 × 105 cells/well) were seeded into a 12-well culture plate and were allowed to grow overnight to reach confluence in DMEM (Singh and Mijakovic, 2022). The monolayer was then scratched using a pipette tip, washed with PBS twice to remove floating cells, and treated with control media. After the incubation period of 24 and 48 h, the cells migrated into the scratched area were photographed under a phase-contrast inverted microscope. The distance that cells had migrated into the cell-free space was measured by ImageJ software. The width of each migrated area was used to calculate the relative proportion wounded at time zero.

#### 2.6.4 Apoptosis assay

To harvest the cells for apoptosis, the corresponding procedure in accordance with cell cycle analysis was conducted up to centrifugation. The Annexin V-FITC (fluorescein isothiocyanate) kit from BD Pharmingen was used for the revelation of apoptosis happening in MCF-7 cells treated with GM-AgNPs and standard in contrast to the control. To bring about the reaction, cells were released in 5 µL of Annexin V-FITC dye protein and agitated, after which 5 µL of PI and 0.5 mL of binding buffer (1×) were added (Giridasappa et al., 2021). They were straight away fed into cell sorting and then flow cytometry, from which only 10,000 cell singlets were gated to distinguish cells undergoing early apoptosis, late apoptosis, and necrosis picked up from Annexin V-PI.

#### 2.6.5 Cell cycle assay

To recognize the arrest of cells at a specified phase, cell cycle analysis had to be executed with the method as discussed by Mousavi-Khattat et al. (2022). The materials were processed within a BD-FACSCalibur flow cytometer (BD Biosciences, San Jose, CA) equipped with laser and PI (propidium iodide) fluorochrome incident at 585 nm by capturing 10,000 events. The analysis was carried out on a 6-well plate using 2 × 105 cells/mL of the MCF-7 cell line dosed with GM-AgNPs. Before staining, the cells were retrieved from the incubator, counted for their number, and placed back inside the incubator to allow cells to grow on the new substratum provided. After treatment with GM-AgNPs, cells were incubated at 37°C for 24 h with 5% CO_2_ supply and then trypsinized to be washed with phosphate-buffered saline (PBS) of 1× concentration. Cells were gathered by centrifugation at 4,000 rpm, washed twice with cold PBS, and then fixed for 30 min using ice cold 70% ethanol. From the BD Cycletest Plus DNA reagent kit, sheath fluid containing PI of 400 µL with 0.05 mg/mL RNaseA was added to the cells and left to incubate for few minutes in the dark. Then, the outcome was analyzed using flow cytometry provided with CellQuestTM Pro version 6.0 software and observed for the cells varying in stages of interphase of the cell cycle.

#### 2.6.6 Caspase-3 assay

The apoptotic protein caspase-3 expression was ascertained in MCF-7 control cells as well as in GM-AgNP-administered MCF-7 cells. During the particularized stage of apoptosis in tumor cells, this marker protease gets promoted and is used as evidence to study their expression levels. This procedure also involves fixing cells as performed during cell cycle analysis, then immersing them in cytofix buffer of about 500 µL for few minutes, and washing them using bovine serum albumin. Subsequently, these cells were treated with a caspase-3 antibody (20 µL) and left to incubate for 30 min in the dark. At last, they were washed with 1× PBS in addition to sodium azide (0.1%) and fed into flow cytometry for the results to be examined (Noorbazargan et al., 2021).

#### 2.6.7 RT-PCR analysis

To measure the expression pattern of certain pro/antiapoptotic genes, RT-PCR was performed using the QuantiTect SYBR Green PCR Kit (QIAGEN) via the ABI PRISM7900HT sequence detection system (Applied Biosystems, Foster City, CA, United States). Real-time PCR cycle parameters included 10 min initial denaturation at 95°C followed by 40 cycles involving denaturation at 95°C for 15 s, annealing at 600°C for 20 s, and elongation at 72°C for 20 s. The sequences of the specific sets of primers for various apoptotic genes, such as Bax and Bcl-2, used in this study have been taken as reported elsewhere (Giridasappa et al., 2021). Real-time PCR experiments were performed in triplicate, and data are expressed as the mean of at least three independent experiments.

#### 2.6.8 Western blot analysis

After the treatment of AgNPs for 12 and 24 h, total cellular proteins were prepared using RIPA lysis buffer (20–188 Merck Millipore, Germany) (included protease inhibitor cocktail) from MCF7 cells (Praburaman et al., 2016). The protein concentrations were established by the bicinchoninic acid assay (71285-Merck Millipore, Germany). An equal amount of protein was separated by 12% polyacrylamide gels and then transferred onto PVDF membranes (sc-3723, SantaCruz, United States). The membranes were blocked with 2.5% BSA at 4°C overnight and then incubated with specific primary antibodies Bax (sc-7480) and Bcl-2 (sc-7382) (SantaCruz, United States). After washing with TBST (containing 0.1% Tween 20) three times, the membranes were incubated with the corresponding HRP-conjugated secondary antibodies in TBST at 37°C for 1 h. The protein β-actin (sc-47778) was used as a housekeeping control for normalization. Finally, the expression levels of proteins were visualized and analyzed using ImageJ software.

### 2.7 Statistical analysis

The above experiments were implemented with three trials. Student’s *t*-test was used to perform comparison between two groups in *in vitro* assays. In all the groups, differences were considered statistically significant among groups with *p* < 0.05. Statistics were performed using GraphPad Prism 8.0.

## 3 Results

### 3.1 Synthesized nanoparticles

**FIGURE 1:**
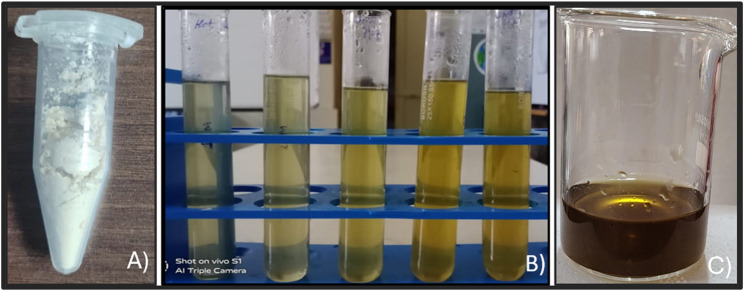
**(A)** Glycine max extract. **(B**) Optimization of GM-AgNPs. **(C)** Final synthesized GM-AgNPs.

The silver nitrate solution was colorless and the GME was white (Figure 1A). After the addition of the GME to the silver nitrate, the solution becomes greenish. During the optimization to different temperatures, the color gradually changed (Figure 1B) and finally turned dark brown (Figure 1C). The color change confirmed the reduction in silver nitrate to silver nanoparticles.

### 3.2 Characterization of the nanoparticles

#### 3.2.1 UV–Vis spectral analysis

**FIGURE 2:**
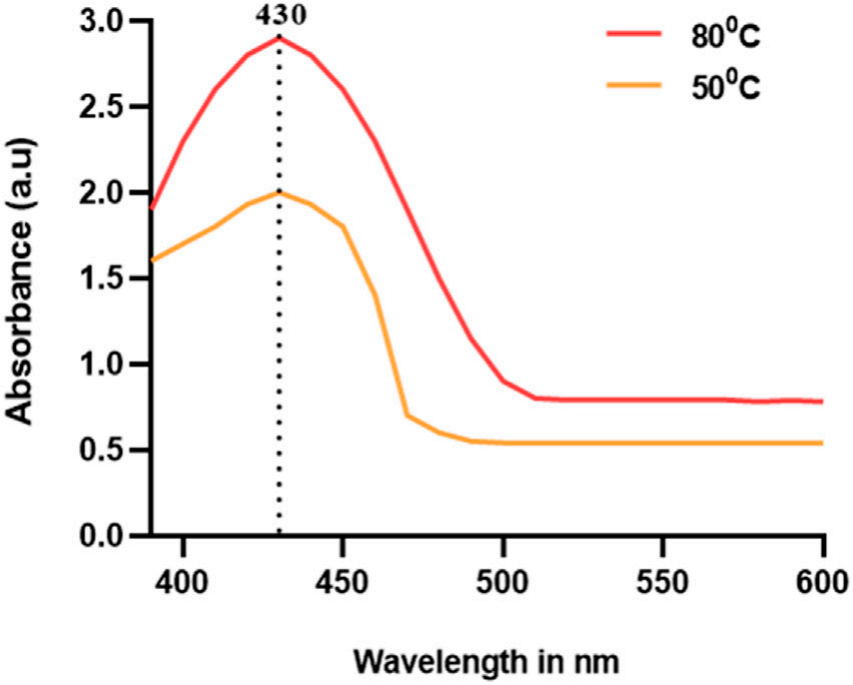
UV–Vis absorbance spectra of phytofabricated AgNPs using extracts of G. max (GM-AgNPs).

UV–Vis spectroscopic analysis is the compulsory required method for the confirmation of metal nanoparticles in the liquid media by measuring the optical absorbance spectra (Kalita et al., 2017). The size and aspect ratio of these NPs have a significant impact on the wavelength of light absorbed. The vivid hues arise from the vibration of conduction electrons on the nanoparticle surface, which is triggered by a certain light wavelength. This effect is explained as surface plasmon resonance. Surface plasmon resonance gives noble metal nanoparticles, like silver, gold, platinum, and copper, distinctive optical characteristics (SPR) (Liang et al., 2012). Because of the near proximity of the conduction and valence bands of metals, which permit free electron mobility between them, the phenomenon is only seen in metal nanoparticles. As a result, the SPR absorption peak is a distinctive feature of synthesized noble metal nanoparticles (Kvítek et al., 2013). In the present experiment, the color shift indicating the presence of Ag nanoparticles was further defined and seen using a UV–Vis spectrophotometer at 500°C and 800°C temperatures (Figure 2). The intense SPR peaks were observed at 430 nm, which was the characteristic peak of AgNPs, and the distinctive UV–Vis range for AgNPs was between 420 and 470 nm. Our results were comparable to those of previously reported green synthesized AgNPs. The UV–Vis spectral analysis confirmed the formation of AgNPs with a distinctive SPR peak at 430 nm, indicating successful synthesis. The optical properties of metal nanoparticles are due to surface plasmon resonance, which is commonly seen in noble metal nanoparticles like silver (Ashraf et al., 2016; Jose et al., 2022).

#### 3.2.2 FTIR spectra

**FIGURE 3:**
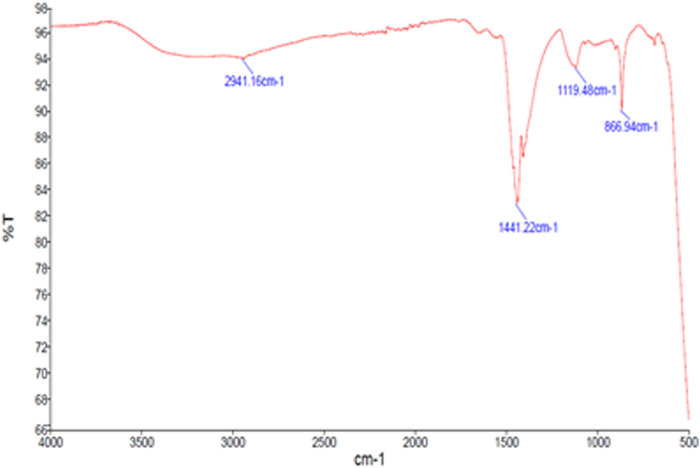
FTIR spectrum of GM-AgNPs.

AgNPs’ surface chemistry has been characterized via FTIR spectroscopy. This technique is utilized to identify the functional groups that are present on NP surfaces (Patra and Baek, 2015). The reducing and capping agents involved in the biosynthesis of AgNPs are demonstrated by the surface-bound functional groups. The capping agents can stabilize the AgNPs (Ferreira et al., 2023). The transmittance values of IR radiation are used to understand the functional groups and chemical bonds of the present phytochemicals. Figure 3 depicts the major wavelengths of IR spectra of synthesized GM-AgNPs. They revealed the presence of major peaks at 2,941.16, 1,441.22, 1,119.48, and 866.94 cm^−1^. The peak at 2,941.16 cm^−1^ represented H–C–H symmetric and asymmetric stretches, and the peak at 1,441.22 cm^−1^ represented H–C–H bends, both of which correspond to the presence of the alkane group. Similar distinguishable peaks are presented by different AgNPs in the previous studies (Devaraj et al., 2013; Wan et al., 2020).

FTIR analysis identified specific functional groups involved in the synthesis, confirming the role of GME phytochemicals as reducing and capping agents. The presence of various functional groups on AgNP surfaces is a common feature in green synthesis, facilitating nanoparticle stabilization. With respect to the phytochemicals in the extract, the major peaks of the FTIR report correspond to the presence of phenols, tannins, carbohydrates, and steroids. These phytochemicals may facilitate the synthesis of AgNPs by acting as reducing and capping agents. Hence, FTIR spectroscopy is an easy and suitable way to ascertain how plant extracts contributed to the current study’s reduction of silver nanoparticles.

#### 3.2.3 XRD patterns

**FIGURE 4:**
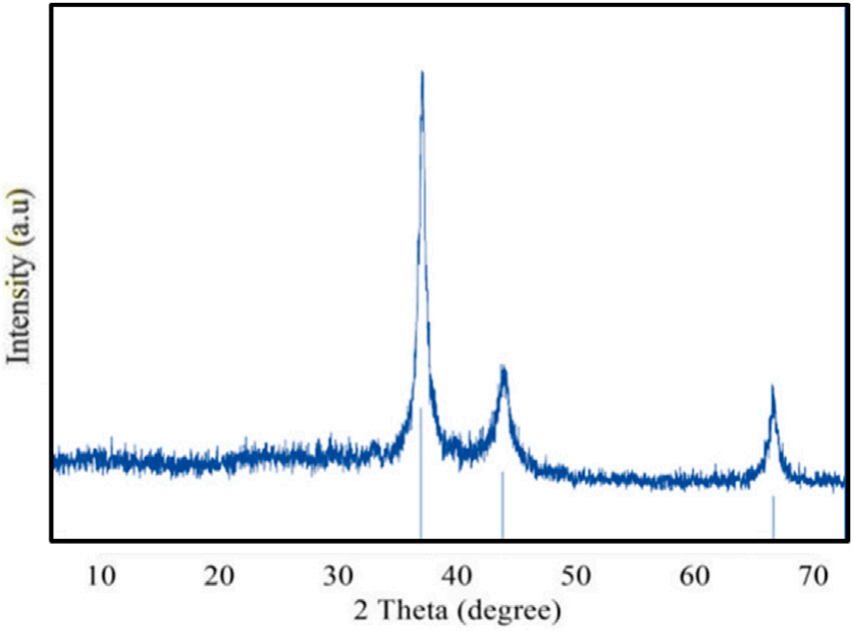
X-ray diffractogram of GM-AgNPs.

X-ray diffraction analysis is used to identify the chemical composition and crystalline structural details of the synthesized nanoparticles (Raja et al., 2022). This study enables to predict the distinct crystallographic structure and particle size of the nanoparticle. The findings demonstrated that silver nanoparticles were present in the nanometric range (Figure 4). The average particle size was calculated using the Debye–Scherrer formula based on the prominent peaks observed at 38.4° and 64.7°. The XRD patterns exhibited three main diffraction peaks and were verified by the TEM–EDX image. The XRD peak patterns demonstrated how the nanoparticle size had a significant effect, emphasizing the need to control the particle size during synthesis. XRD analysis confirmed the crystalline nature of the GM-AgNPs with distinct diffraction peaks, indicating successful nanoparticle synthesis. XRD patterns are typically used to determine the crystalline structure and particle size of nanoparticles.

#### 3.2.4 SEM/TEM/EDX analysis

**FIGURE 5:**
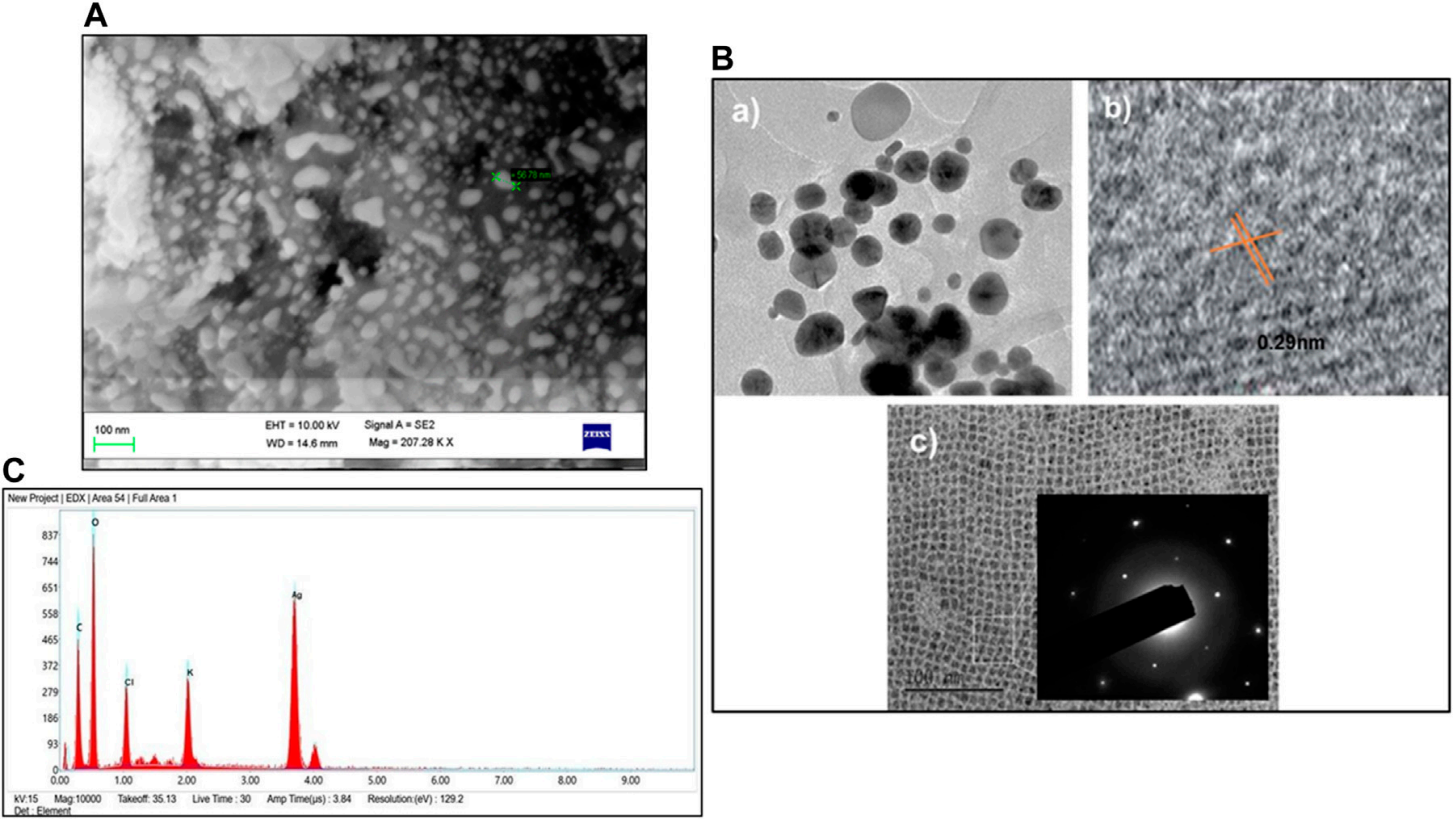
**(A)** SEM analysis of GM-AgNPs. **(B)** TEM analysis of GM-AgNPs: (a) TEM micrographs showing spherical silver nanoparticles, (b) HRTEM image showing interplanar distance of 0.29 nm, and (c) SAED patterns showing crystallinity of AgNPs. **(C)** EDX of GM-AgNPs.

The shape of GM-AgNPs is critical in determining their physicochemical properties and possible uses. SEM and TEM are the advanced techniques employed to analyze the morphology of the sample. TEM analysis shows the high resolution and allows to detect each nanoparticle through their interactions with the electron beam and creates images on the photographic plate (Malatesta, 2021). SEM analysis revealed the equal distribution of GM-AgNPs and exhibited their spiral shape with the size range of 5–50 nm (Figure 5A). TEM analysis further detailed the polycrystalline structure of the nanoparticle with distinct boundaries. Their crystallinity was observed through the selected area electron diffraction pattern and lattice pattern of the GM-AgNPs, as shown in Figure 5B, depicting the size of 10 nm. SEM and TEM analyses confirmed the spherical shape and polycrystalline nature of the GM-AgNPs. SEM and TEM analyses provide detailed insights into the morphology and crystalline structure of nanoparticles.

Further elemental analysis was conducted via energy-dispersive X-ray spectroscopy (EDX) (Nasrollahzadeh et al., 2019), which showed a strong absorption peak at 3 eV (Figure 5C). The EDX analysis shows the percentages of elemental compositions such as carbon (C): 29%, oxygen (O): 48.2%, chloride (Cl): 6.4%, potassium (K): 3.7%, and silver (Ag): 11.9%. The other chemicals act as capping organic agents that are bound to the surface of silver nanoparticles. This peak signifies the presence of silver, which is a primary component of GM-AgNPs. This morphological analysis of data helps understand the properties of synthesized nanoparticles and promises the potential of GM-AgNPs for biological applications, whereas EDX analysis validated the elemental composition, highlighting the presence of silver.

### 3.3 Antibacterial activity

**FIGURE 6:**
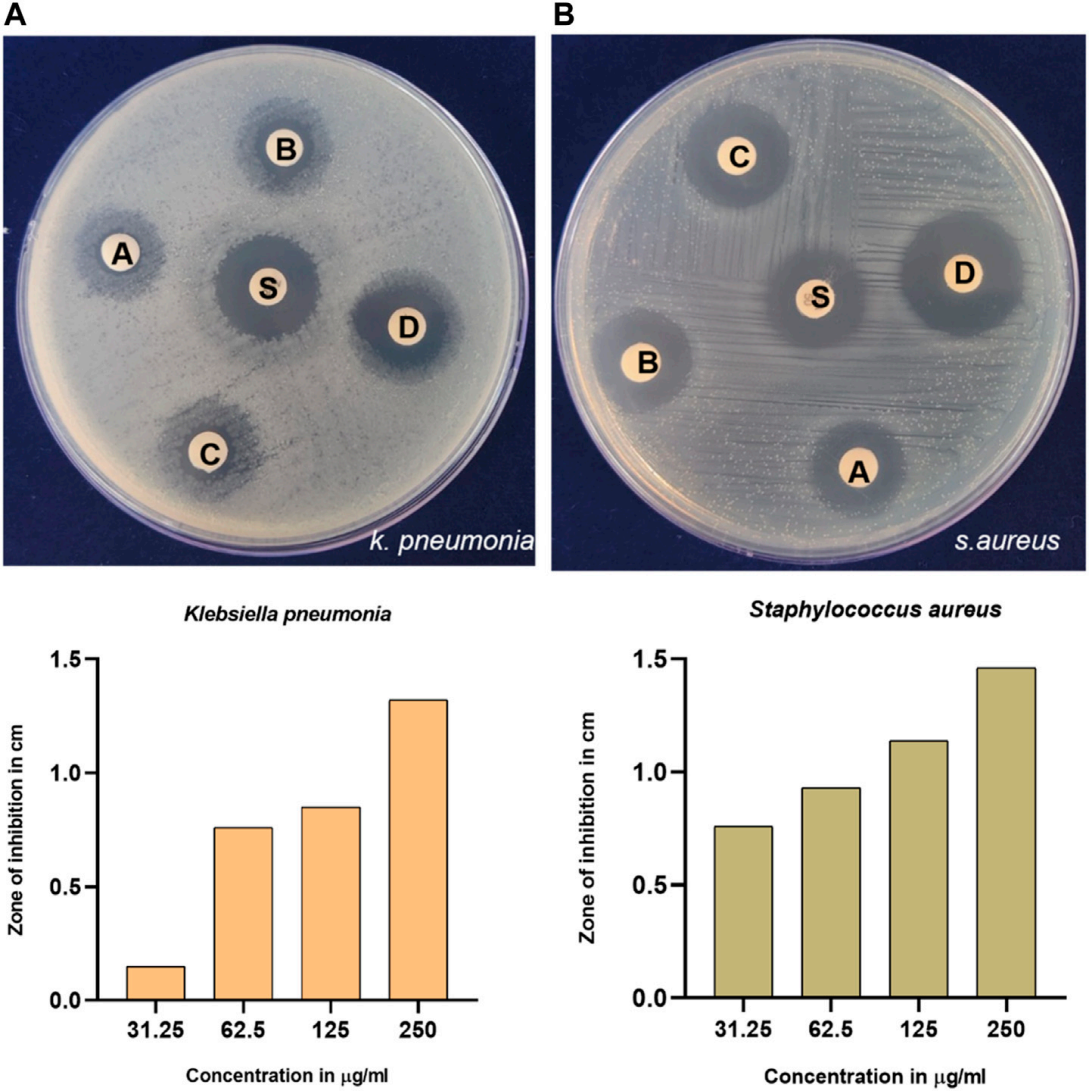
Antimicrobial activity of synthesized GM-AgNps against **(A)** *K.* pneumonia and **(B)** *S. aureus* (disc labeling: A = 31.25 μg/mL; B = 62.5 μg/mL; C = 125 μg/mL; D = 250 μg/mL; and S = gentamycin of 10 mcg). **(B)** Concentration-dependent antibacterial activity of GM-AgNPs.

**FIGURE 7:**
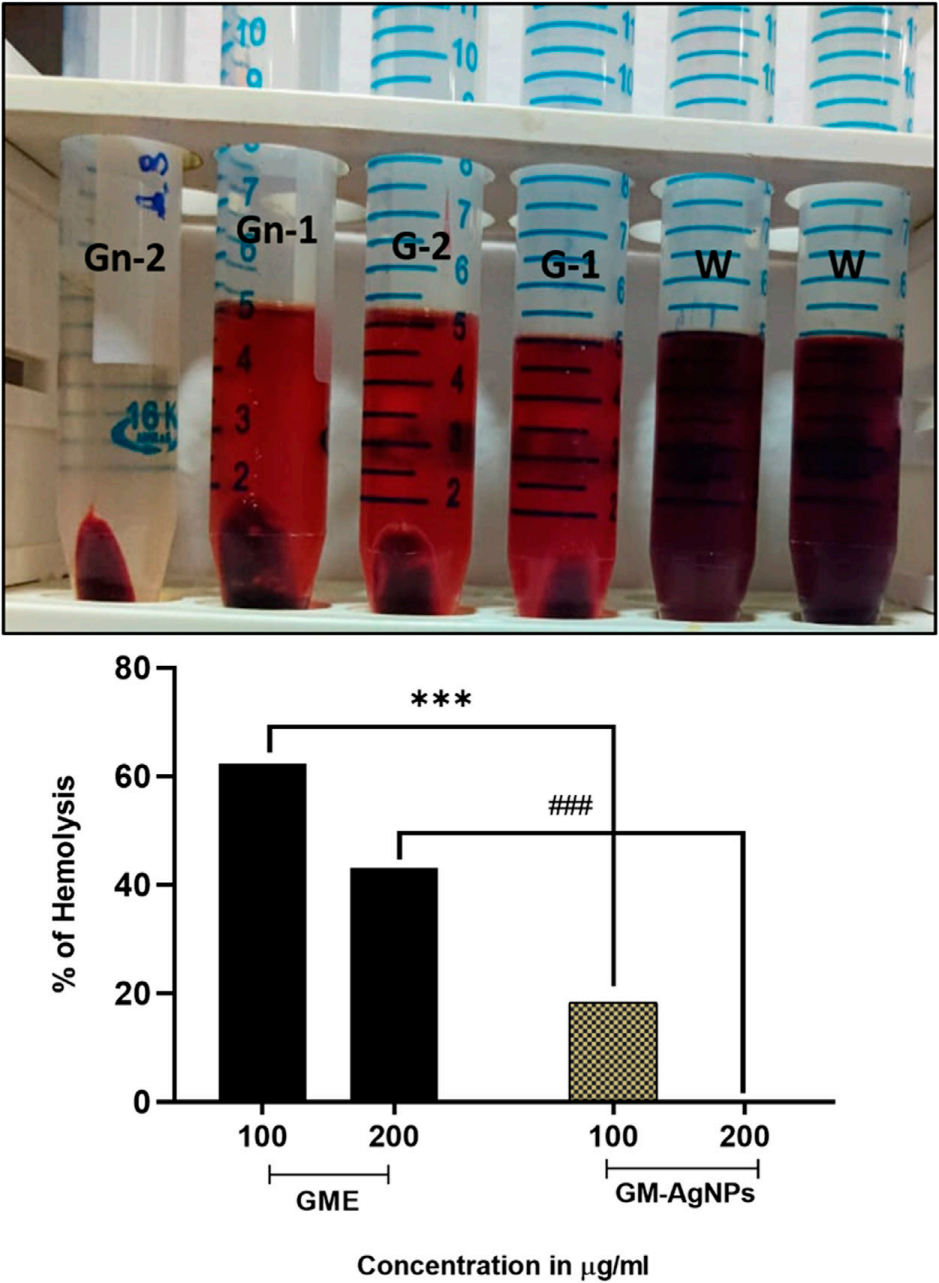
Percentage of hemolysis by GME and GM-AgNPs. Unpaired *t*-test was used to identify significant differences in two-group comparisons. The tests were two-tailed, and data are expressed as mean ± SEM (n = 3), ***p < 0.001 between GME and GM-AgNPs at 100 μg/mL (−43.97 ± 0.3232) and ###*p* < 0.001 at 200 μg/mL (−42.94 ± 0.5207).

Infections with bacteria are frequently the cause of illnesses in humans. Transmittable infections can be altered as risk factors for cancer (Liardo et al., 2021). Hence, to explore the biological activity of synthesized GM-AgNPs, antimicrobial activity was analyzed. Among many bacteria, *K. pneumonia* and *S. aureus* have a major role in cancer etiology and secondary infection details in cancer patients (Zembower, 2014; Wong and Evans, 2017; Strakova et al., 2021; Yusuf et al., 2023). Thus, in the present work, synthesized AgNPs were tested against the Gram-positive bacteria *S. aureus* and the Gram-negative bacteria *K. pneumonia*. Gentamicin was used as a standard drug. At a higher concentration of 250 μg/mL, both the bacterial plates exhibit a higher zone of inhibition than a standard drug. As shown in Figures 6, 7, antimicrobial test results show the diameter of the zone of inhibition in different concentrations of synthesized AgNps against respective organisms. Gentamicin at 10 mcg showed a 1.32 cm of zone of inhibition against *S. aureus* and 1.41 cm against *K. pneumonia*.

### 3.4 Hemolysis

**FIGURE 8:**
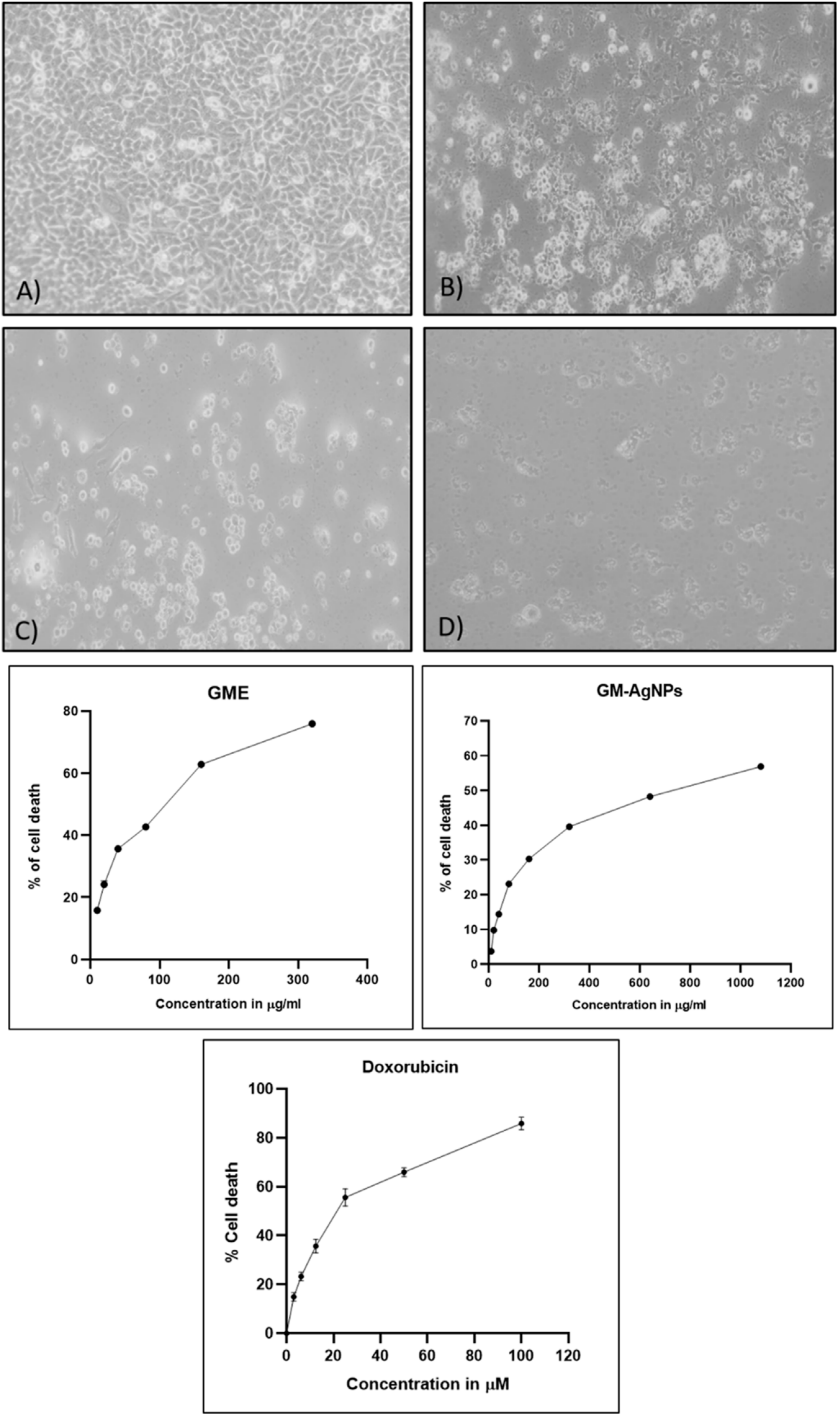
**(A)** Cytotoxicity analysis of samples treated on MCF-7 cell lines: **(a)** untreated, **(b)** GME, **(c)** AgNO3, and **(d)** GM-AgNPs. **(B)** IC50 calculation of samples GME and GM-AgNPs with standard doxorubicin. Data represent mean ± SD (n = 3).

To determine the biocompatibility of the synthesized AgNPs, the anti-hemolysis factor was analyzed (Chahardoli et al., 2021). Anti-hemolysis was effectively analyzed using donor blood in comparison with the GME on varied concentrations. With the increase in the 200 μg/mL concentration, the GM-AgNPs showed no hemolysis, whereas CME showed around 40% hemolysis at 200 μg/mL concentrations (Figure 8). These data deduce the biocompatibility and medicinal application of GM-AgNPs inside the biological system. Similar reports have been conveyed in previous studies involving green synthesized nanoparticles (Ashokraja et al., 2017; Chahardoli et al., 2021; Liaqat et al., 2022).

### 3.5 Anticancer activity

After confirmation of the biosynthesized AgNPs relating to their quality and stability, their biological activities were analyzed in relation to their application as biomedicine.

#### 3.5.1 MTT assay

The cytotoxicity assay is a common method used to assess the toxic effects of compounds or extracts on cells (McGaw et al., 2014). This assay helps determine the viability and survival of cells in the presence of the test substance. The MTT (3-(4,5-dimethylthiazol-2-yl)-2,5-diphenyltetrazolium bromide) reaction is a critical step in the cytotoxicity assay that enables the measurement of cell viability or proliferation. The MTT assay relies on the ability of viable cells to reduce MTT into formazan crystals, which can be quantified spectrophotometrically. It is essential to note that the MTT assay has limitations, including potential interference from specific compounds or precipitation of the dye under certain conditions. Here, GM-AgNPs were found to have cytotoxic effects against MCF-7 cell lines in a concentration-dependent manner with the IC50 value of 74.04 μg/mL in comparison with the GME extract with the IC50 value of 145.11 μg/mL (Figures 8). This 50% cytotoxic concentration was used for further experiments in this study. Earlier studies report the same type of results studying the effect of green synthesized AgNPs in MCF-7 cells (Khorrami et al., 2018). The data clearly interpret the efficiency of GM-AgNPs against breast cancer cell lines in comparison with the crude extract, as GM-AgNPs are more cytotoxic to the MCF-7 cells at very low concentration than the GME. Doxorubicin at 24.82 µM concentration showed 50% of cell death.

#### 3.5.2 Cell migration assay

**FIGURE 9:**
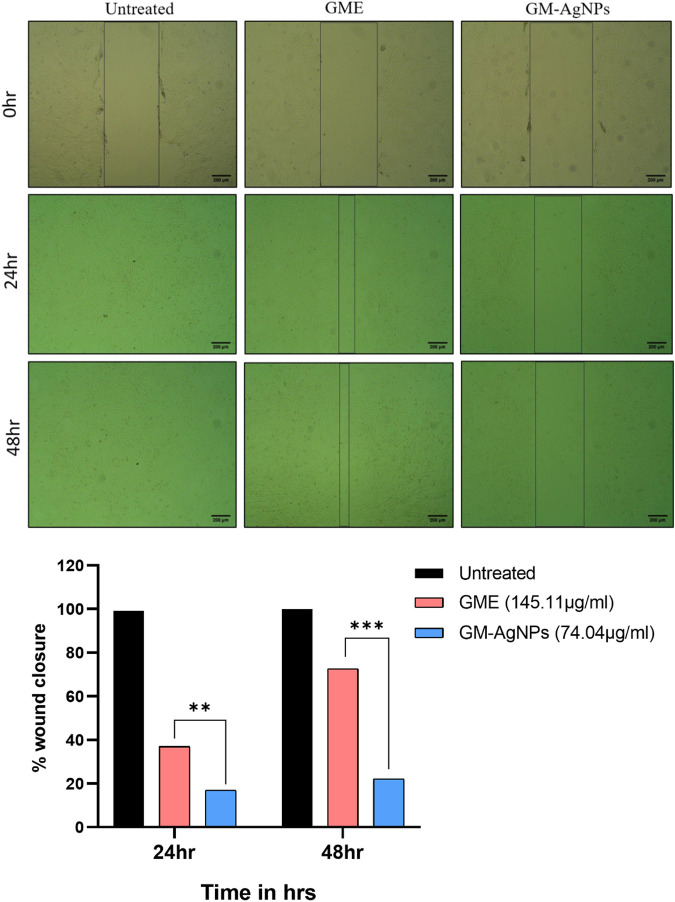
Cell migration analysis among treatment groups (IC50) at 24 and 48 h on MCF-7 cell lines. Unpaired *t*-test was used to identify significant differences in two-group comparisons. The tests were two-tailed, and data are expressed as mean ± SEM (n = 3), **p < 0.002 between GME and GM-AgNPs (−19.94 ± 0.7840) at 24 h and ***p < 0.001 (−50.47 ± 1.410) at 48 h.

Cancer cells separate from the original tumor site, infiltrate adjacent tissues, enter the bloodstream or lymphatic system, and create secondary tumors at other locations throughout the body through the intricate process of tumor metastasis (Fares et al., 2020). The ability of cancer cells to migrate is a crucial quality that underlines their capability to disseminate and colonize new tissues, and it facilitates this complex chain of events. Despite the GM-AgNPs’ ability to cytotoxicity, their potency to inhibit cancer cell migration has been explored using the scratch assay method. The present experimental data of GM-AgNPs in comparison with the extract at its IC50 concentration show significant inhibition of cell migration at 24 and 48 h, whereas the GME and control cells show more than 70% and 100% cell migration, respectively (Figure 9).

#### 3.5.3 Apoptosis

**FIGURE 10:**
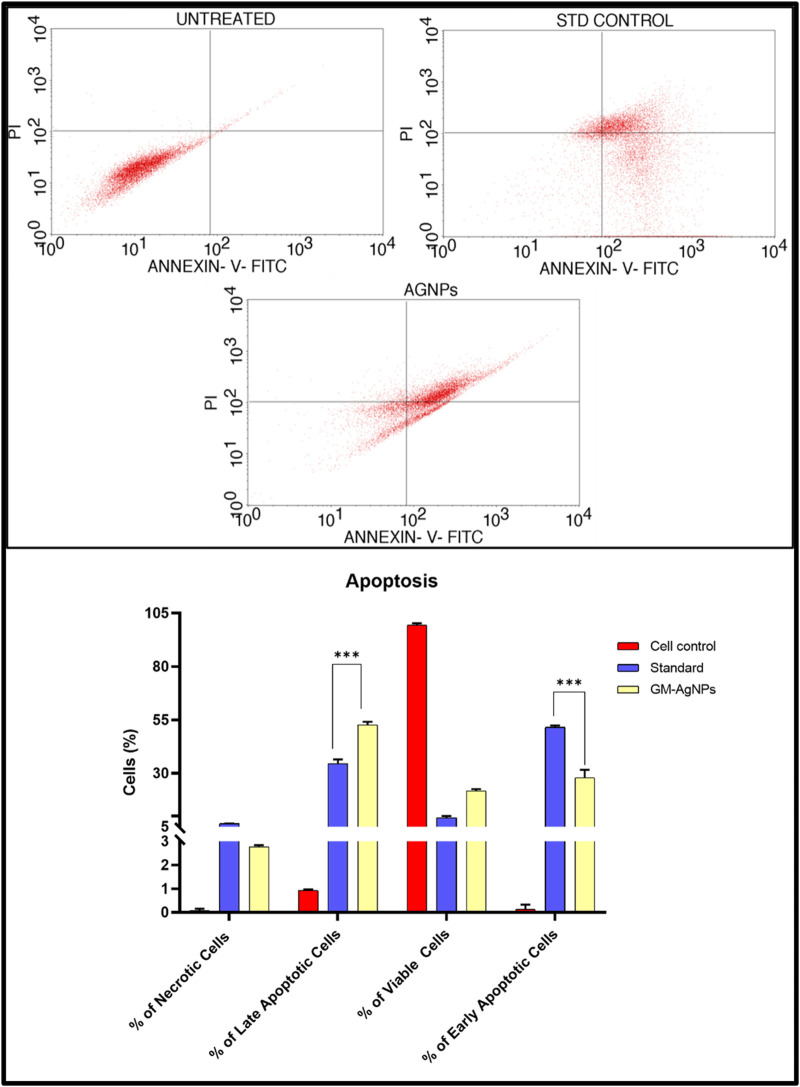
**(Above)** Annexin V-PI expression study for the apoptosis of GM-AgNPs against MCF-7 cells using BD FACSCalibur. **(Below)** Graphs present the percentage of apoptotic cells. Values represent the mean of three experiments ± standard deviation (SD). Unpaired *t*-test was used to identify significant differences in two-group comparisons. The tests were two-tailed, and data are expressed as mean ± SEM (n = 3), 17.89 ± 1.108 and −11.13 ± 0.9540 between standard and GM-AgNPs late apoptosis and early apoptosis, respectively (*p* < 0.0002).

Apoptosis, which is a fundamental process in cell biology, is essential for maintaining tissue homeostasis, development, and the elimination of damaged or unwanted cells from multicellular organisms (Elmore, 2007). The Annexin V/PI staining assay is a versatile and successful technique for identifying apoptotic cells by focusing on phosphatidylserine externalization on the cell membrane. During apoptosis, phosphatidylserine, which is normally found on the inner leaflet of the plasma membrane in healthy cells, becomes exposed on the cell surface (Chaurio et al., 2009). Annexin V, which is a calcium-dependent phospholipid-binding protein with a high affinity for phosphatidylserine, acts as a sensitive marker for this early stage of apoptosis. This technique, when paired with PI, a membrane-impermeable dye that stains necrotic and late-stage apoptotic cells, enables the differentiation of various cell populations based on membrane integrity (Vermes et al., 1995). Hence, in the present study, the Annexin V/PI staining test was performed to confirm apoptosis. The assay indicated apoptosis in cancer cells treated for 24 h to GM-AgNPs (74.04 μg/mL) in comparison with control. As shown in Figure 10, the cells treated with GM-AgNPs show more of late apoptotic cell population of 50.92% and early apoptotic cell population of 23.56%. Variations in the number of viable cells suggest that GM-AgNP-induced anticancer actions cause the cell to undergo apoptosis. Previous studies on green synthesized AgNPs have shown a similar effect, which justifies our data of antitumor activity relating to the apoptotic pathway (Ullah et al., 2020a; Ullah et al., 2020b Bin-Jumah et al., 2020).

#### 3.5.4 Cell cycle analysis

**FIGURE 11:**
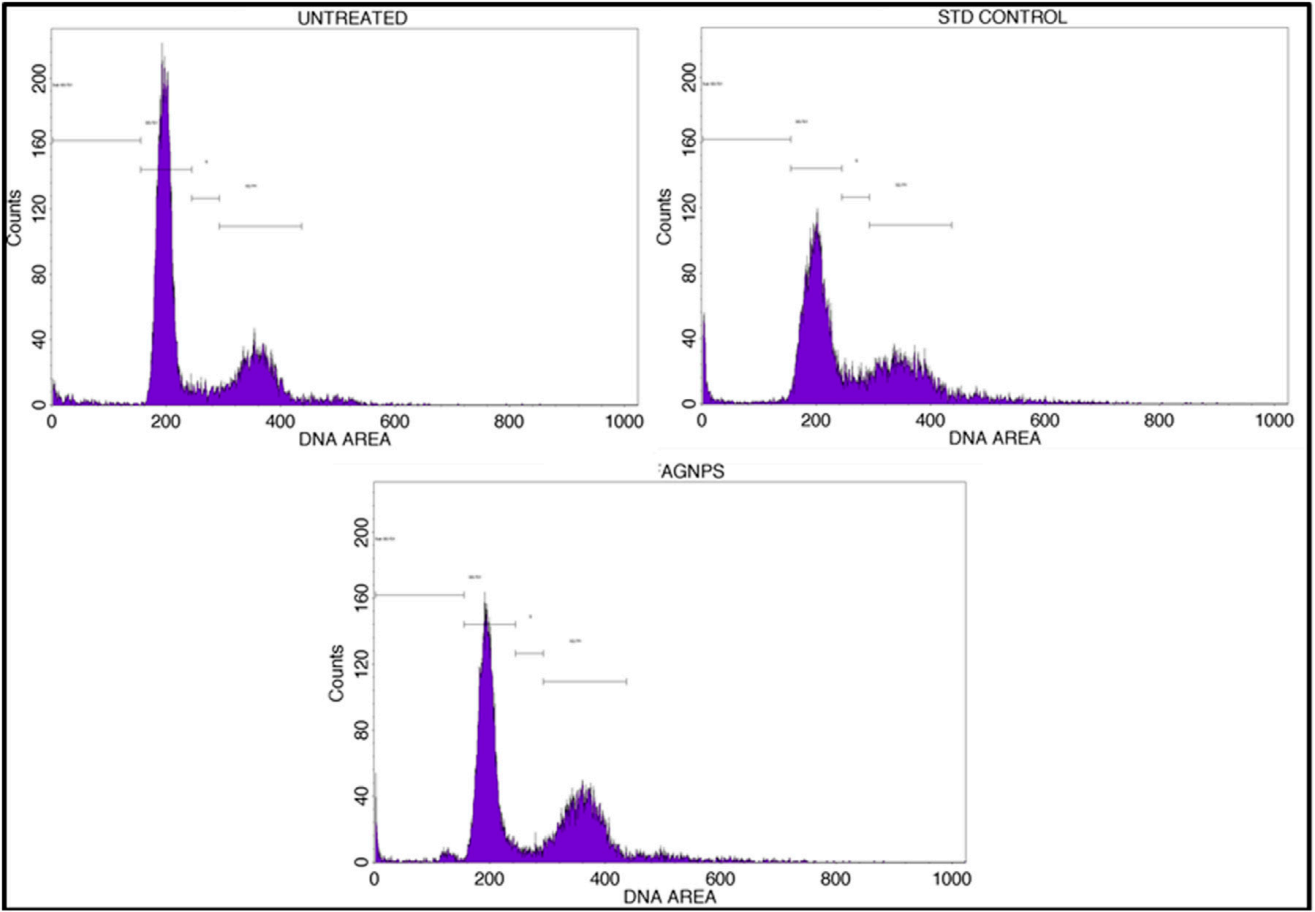
Annexin V-PI expression study for cell cycle of GM-AgNPs against MCF-7 cells using BD FACS Calibur.

A cell cycle arrest test is a laboratory technique used to look into how different substances or external circumstances affect the control of the cell cycle (Kari et al., 2022). The cell cycle is a highly orchestrated process through which eukaryotic cells replicate and divide. It consists of distinct phases, including G1 (Gap 1), S (Synthesis), G2 (Gap 2), and M (Mitosis), with checkpoints that ensure the accuracy and integrity of cellular division. An assay for cell cycle arrest primarily aims to evaluate a substance’s or condition’s capacity to stop or postpone a cell’s progression through a given checkpoint in the cell cycle. It aids in the discovery of substances that may be able to stop cancer cells from growing out of control by stopping the advancement of their cell cycle. Based on the profile of the cell cycle with flow cytometry, treatment with GM-AgNPs shows induction of apoptosis as the cell load has drastically decreased in the sub-G0/G1 (growth phase) phase (48.19%) in comparison with the control (65.99%) and standard (52.09%). Percentages of cell counts in each cycle phase are shown in Table 1; Figure 11. Several other nanoparticles with the potency of inducing apoptosis have shown similar flow patterns in previous studies (Kim et al., 2013; Estevez et al., 2014).

**TABLE 1:** Percent of cells which got arrested after the drug treatment in different stages of their life cycle and overlay of the results plotted in the stacked graph.

S. no	Cell cycle stage	Cell control	Standard	GM-AgNP
1	Sub G0/G1	3.13	5.36	11.21
2	G0/G1	65.99	52.03	48.19
3	S	4.51	7.07	6.77
4	G2/M	26.37	35.54	33.83
	Total events selected	10,000	10,000	10,000

#### 3.5.5 Caspase-3 expression

**FIGURE 12:**
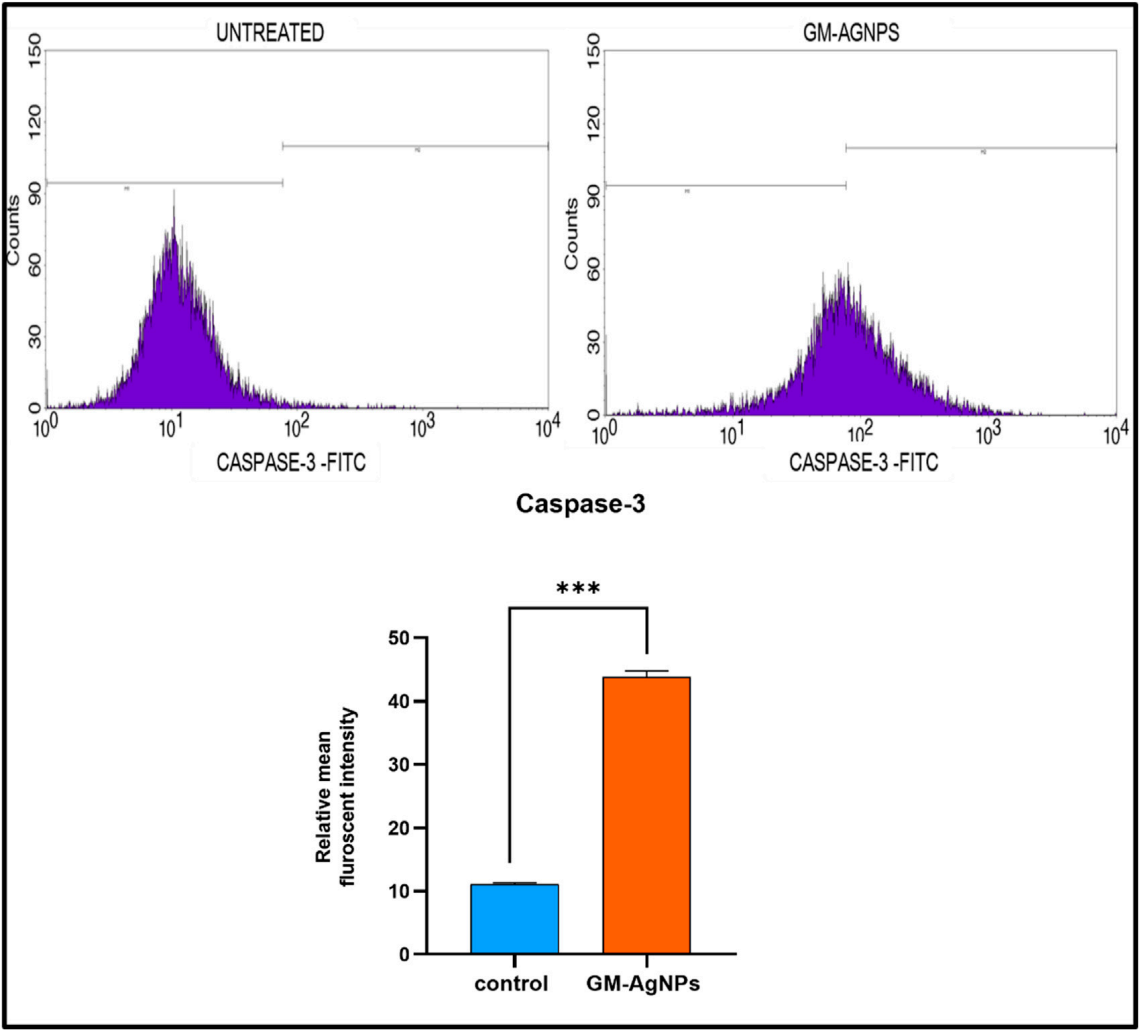
Above Caspase 3-FITC density plot and CASPASE 3-FITC histogram of the gated MCF7 singlets distinguishing cells at the M1 and M2 phases in untreated and GM-AGNPs cells. Below Relative mean fluorescent intensity of caspase-3 expression. Values represent the mean of three experiments ± standard deviation (SD). Unpaired *t*-test was used to identify significant differences in two-group comparisons. The tests were two-tailed, and data are expressed as mean ± SEM (n = 3). Significance of ***p < 0.002 between control and GM-AgNPs (32.79 ± 0.5635).

Caspase-3 is a protease enzyme that plays a central role in the execution phase of apoptosis (Porter and Jänicke, 1999). The activation of caspase-3 leads to the cleavage of various cellular proteins, ultimately resulting in cell death. The activation of caspase-3 is essential to induce apoptosis in cancer (Thomsen et al., 2013; Boice and Bouchier-Hayes, 2020). To explore the potency of GM-AgNPs relating to the induction of apoptosis by caspase-3 activation, their expression was evaluated using flow cytometry analysis. The experimental results showed a substantial increase in caspase-3 expression with a relative intensity of 44.91, whereas the control showed a relative intensity of 11.22 (Figure 12). Similar mechanistic data were noted with different plant extracts and Ag-NPs against MCF-7 cell lines (Tang et al., 2019; Chang et al., 2021).

#### 3.5.6 PCR analysis

As observed from the previous experimental reports, it was confirmed that GM-AgNPs trigger the apoptosis of the cancer cells. Hence, further major apoptotic markers, namely, Bax and Bcl-2 expression studies, were performed (Klimentova et al., 2021). Bax (Bcl-2-associated X protein) and Bcl-2 (B-cell lymphoma 2) are two key proteins involved in the regulation of apoptosis, which is a tightly controlled process of programmed cell death. These proteins are part of the Bcl-2 family, which includes both pro-apoptotic and antiapoptotic members that interact to determine the fate of a cell (Qian et al., 2022; Kaloni et al., 2023). Bcl-2 is an antiapoptotic protein that inhibits cell death and promotes cell survival. It is also located on the mitochondrial outer membrane and other cellular membranes (Kale et al., 2018). Bcl-2 prevents the release of cytochrome c and other pro-apoptotic factors from the mitochondria by inhibiting the actions of pro-apoptotic proteins like Bax (Hardwick and Soane, 2013), whereas Bax is a pro-apoptotic protein, meaning it promotes cell death when activated. It is mainly located in the cytoplasm of cells but can translocate to the mitochondrial outer membrane in response to pro-apoptotic signals. Bax undergoes conformational changes, forming channels or pores that allow the release of cytochrome c and other pro-apoptotic factors from the mitochondria (Eskes et al., 2000). The release of cytochrome c triggers a cascade of events, including the activation of caspases, which are proteases that carry out the execution of apoptosis by degrading cellular components (Qian et al., 2022).

Based on the mRNA expression through RT-PCR, it was observed that the decreased expression of bcl-2, an anti-cell death factor, was significantly correlated with the treatment. At 48 h, in comparison with the untreated sample, Bcl-2 showed a considerable decrease, confirming the increased death of treated cells, whereas the apoptotic factor Bax expression increased radically in folds, signifying the initiation of apoptosis. However, the Bax/Bcl-2 ratio was significant higher for GM-AgNPs (>1).

#### 3.5.7 Western blot

**FIGURE 13:**
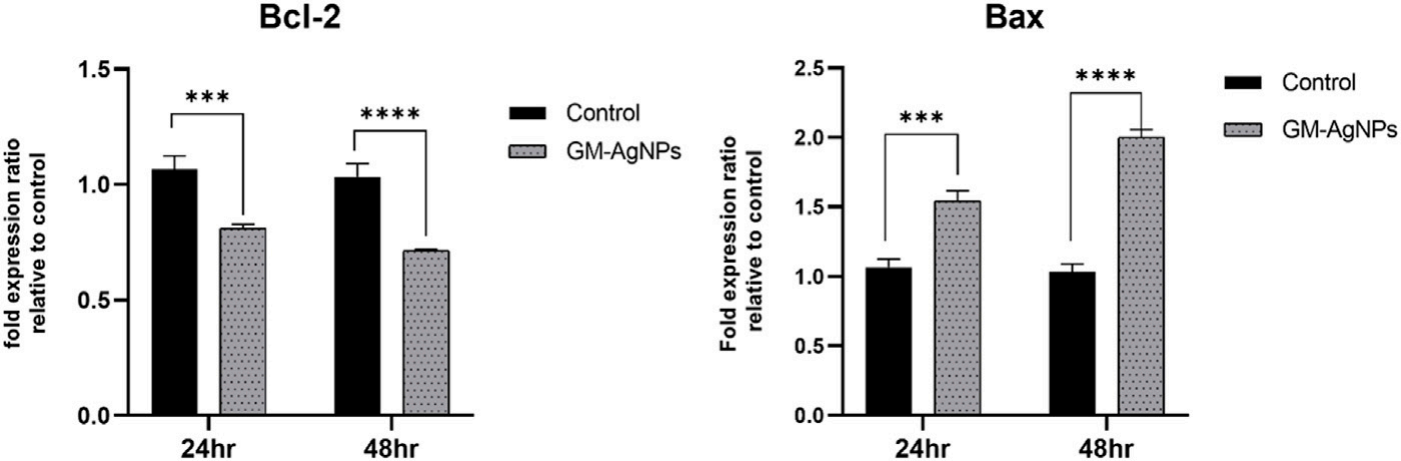
Fold expression ratio of Bcl-2 between control and GM-AgNPs. Values represent the mean of three experiments ± standard error mean. Two-way ANNOVA was applied for multiple group comparisons. ***p = 0.0001 vs. control at 24 h (0.2570 ± 0.034) and ****p < 0.0001 at 48 h s (0.3194 ± 0.034). Fold expression ratio of Bax between control and GM-AgNPs. Values represent the mean of three experiments ± standard error mean. Two-way ANNOVA was applied for multiple group comparisons. ***p = 0.0004 vs. control at 24 h (−0.4767 ± 0.073) and ****p < 0.0001 at 48 h (−0.9667 ± 0.073).

**FIGURE 14:**
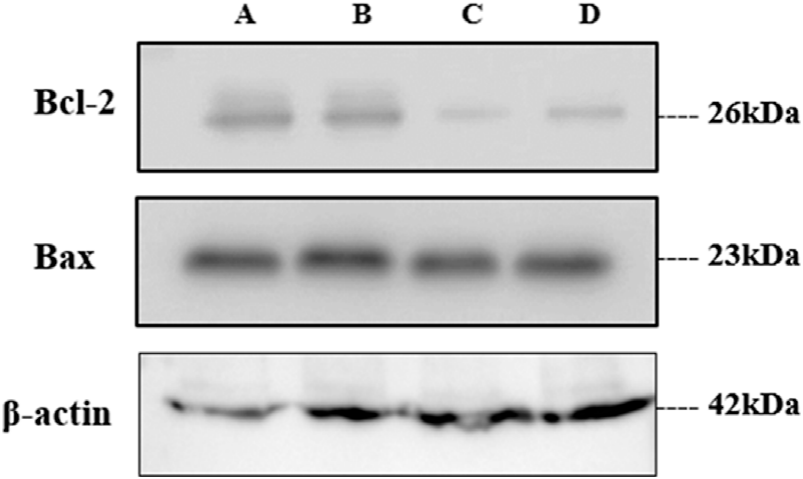
Western blot analysis of treated samples. (**A)** Untreated, **(B)** GME-treated, and **(C, D)** GM-AgNPs at 48 and 24 h

Like the gene expression of Bax and Bcl-2 by RT-PCR, the protein expression of the same was analyzed using the Western blot technique. On treatment with GM-AgNPs for different time intervals, Bcl-2 protein expression was decreased, and the data were well correlated with the gene expression data, whereas in the Western blot analysis, Bax protein expression was not in accordance with the treatment and gene expression data. Bax protein expression had a very less impact on treatment, even at 24 and 48 h (Figures 13, 14).

## 4 Discussion

Soybeans are well known for their nutritional benefits, being rich in bioactive compounds such as isoflavones, proteins, and fibers. They are also readily available, making them a valuable resource for various applications, including the green synthesis of nanoparticles. Green synthesis involves the use of plant extracts to produce nanoparticles, offering an eco-friendly and cost-effective alternative to conventional chemical and physical methods. This approach leverages the natural reducing agents present in plant extracts to convert silver ions into silver nanoparticles. The process is favored for its simplicity, lower toxicity, and biocompatibility. Recently, the green synthesis of silver nanoparticles (AgNPs) using plant-based extracts has garnered significant attention in cancer treatment due to their potent bioactivity and environmentally friendly production methods. Hence, in the present study, soybean extracts (GM) were utilized to synthesize silver nanoparticles (GM-AgNPs), which were then evaluated for their anticancer potency.

The synthesized GM-AgNPs underwent comprehensive characterization using several techniques. UV-visible spectroscopy confirmed the formation of nanoparticles by showing a characteristic absorption peak, usually around 400–450 nm, which is indicative of surface plasmon resonance. Fourier transform-infrared (FT-IR) spectroscopy helped identify the functional groups involved in the synthesis process, whereas scanning electron microscopy (SEM) and transmission electron microscopy (TEM) provided detailed information on the morphology and size of the nanoparticles. Energy-dispersive X-ray (EDX) analysis confirmed the elemental composition of the GM-AgNPs, further validating their successful synthesis.

The antibacterial efficacy of GM-AgNPs was tested using the disc diffusion method, revealing significant activity against *S. aureus* and *K. pneumoniae.* The maximum zone of inhibition was observed at a concentration of 250 μg/mL, comparable to the antibiotic gentamicin. This highlights the potential of the GM-AgNP as an effective antibacterial agent. Several studies explore the antibacterial activity of AgNPs synthesized using various plant extracts, reporting zones of inhibition against *S. aureus* and *K. pneumoniae*, ranging from a few millimeters to over 20 mm depending on factors such as plant source, AgNP concentration, and bacterial strain. Sharma et al. (2008) explored AgNPs from various plants and reported their antibacterial activity against *S. aureus* and *K. pneumoniae*. However, it does not compare the activity with a standard antibiotic. Additionally, the anti-hemolytic activity was assessed *in vitro*, demonstrating that at 200 μg/mL, GM-AgNPs caused no hemolysis, contrasting with the soybean extract alone, which induced 40% hemolysis. This suggests a safety profile for GM-AgNPs in terms of hemolysis. Previous studies on the anti-hemolytic properties of various plant extracts are explored, and some plants exhibited anti-hemolytic activity, whereas others might have hemolytic properties depending on the extract concentration and the specific plant source.

The anticancer properties of GM-AgNPs were evaluated against a breast cancer cell line. The nanoparticles exhibited an IC50 value of 74.04 μg/mL, indicating a moderate level of cytotoxicity. Numerous studies explore plant extracts and plant-derived nanoparticles for their anticancer potential. Khan et al. (2023) also investigated the green synthesis of silver nanoparticles (AgNPs) using *Moringa oleifera* leaf extract and explored their anticancer and antioxidant activities. These studies report a wide range of IC50 values, depending on the plant source, type of cancer cell line, and the specific compounds involved.

Many anticancer drugs target tumor growth and proliferation. However, some drugs also aim to inhibit metastasis, which is a critical step for cancer progression. Several studies explore the effect of various agents on cancer cell migration and invasion, which are key processes in metastasis. Further analysis at the IC50 concentration showed a reduction in cancer cell migration, suggesting the potential to inhibit metastasis. Chatterjee et al. (2017) suggested ROS generation and mitochondrial dysfunction as potential contributing factors. Investigating if GM-AgNPs follow a similar mechanism or have a unique pathway would be valuable. The study highlights cell cycle arrest as an additional mechanism. Understanding the specific molecules or pathways targeted by GM-AgNPs to arrest the cell cycle in the sub G0/G1 phase would provide deeper insights. The mechanism underlying the anticancer activity of GM-AgNPs involved the induction of apoptosis and cell cycle arrest in the sub-G0/G1 phase, with a 48.19% increase. Additionally, the observed changes in Bax and Bcl-2 protein levels support apoptosis induction. Further investigation could quantify the exact changes and explore if GM-AgNPs directly regulate their expression or act through other signaling pathways.

Whereas these findings suggest that GM-AgNPs synthesized from soybean extracts possess significant antibacterial and anticancer activities, making them promising candidates for therapeutic applications, there are potential limitations and future research directions to consider. One limitation is the potential variability in the composition of plant extracts, which could affect the consistency of nanoparticle synthesis and efficacy. Future research should focus on standardizing extraction methods and characterizing the active components in soybean extracts that contribute to nanoparticle synthesis. Additionally, comprehensive *in vivo* studies are needed to confirm the safety and efficacy of GM-AgNPs in clinical settings. Exploring the mechanisms of action in greater detail, including their interactions with cellular pathways and the immune system, would provide deeper insights into their therapeutic potential. Finally, expanding the scope of research to include other types of cancer cells and drug-resistant bacterial strains could further validate the broad-spectrum applicability of GM-AgNPs in biomedical applications.

## 5 Conclusion

In conclusion, the study successfully demonstrated the green synthesis of silver nanoparticles (GM-AgNPs) using *Glycine max* (soybean) seed extracts, highlighting their significant potential in biomedical applications. The synthesis process was optimized and confirmed through multiple characterization techniques, ensuring the stability and uniformity of the nanoparticles. GM-AgNPs exhibited strong antibacterial activity against both Gram-positive bacteria and Gram-negative bacteria, outperforming standard antibiotics like gentamycin, and showed minimal hemolytic activity, indicating their safety for medical use. Furthermore, the nanoparticles demonstrated potent anticancer effects against MCF-7 breast cancer cells, including dose-dependent cytotoxicity, inhibition of cell migration, induction of apoptosis, and cell cycle arrest. These findings underscore the promise of GM-AgNPs as effective antimicrobial and anticancer agents, advocating for further *in vivo* studies and detailed mechanistic investigations to fully harness their therapeutic potential in drug delivery, antimicrobial treatments, and cancer therapy.

## Data Availability

The original contributions presented in the study are included in the article/Supplementary Material; further inquiries can be directed to the corresponding authors.
