# Supplementary material for: Bactericidal, anti-hemolytic, and anticancerous activities of phytofabricated silver nanoparticles of glycine max seeds
Source: Front Chem. 2024 Aug 16;12:1427797. doi: 10.3389/fchem.2024.1427797 (PMC11447554; doi:10.3389/fchem.2024.1427797)
Supplement: Supplementary file 1 [file DataSheet1.docx]

**Supplementary Data**

Anti-bacterial anlysis

| **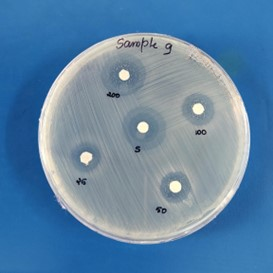**  **A)** | **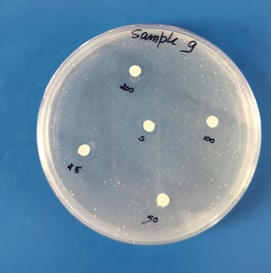**  **B)** |
| --- | --- |

| **Extract** | **Concentration** | ***Pseudomonas aeruginosa*** | ***E.coli*** |
| --- | --- | --- | --- |
| CFEA | 25 µg/ml | 0.25 cm | 0.15 cm |
|  | 50 µg/ml | 0.30 cm | 0.20 cm |
|  | 100 µg/ml | 0.35 cm | 0.25 cm |
|  | 200 µg/ml | 0.61cm | 0.37 cm |
| Tetracycline | | 0.46cm | 1.02 cm |

**DLS to confirm the average size of AgNPs**

**

**

**Fig-5d:** Peaks confirming the average particle size in nanometers by DLS analysis
